# Mutations of uncertain significance in heterozygous variants as a possible cause of severe short stature: a case report

**DOI:** 10.1186/s40348-020-00104-6

**Published:** 2020-09-16

**Authors:** Nami Mohammadian Khonsari, Sahar Mohammad Poor Nami, Benyamin Hakak-Zargar, Tessa Voth

**Affiliations:** 1 School of Medicine, Alborz University of Medical Sciences, Karaj, Alborz Iran; 2 Faculty of Health Sciences, Simon Fraser University, Burnaby, British Columbia Canada; 3 Department of Biomedical Physiology and Kinesiology, Faculty of Science, Simon Fraser University, Burnaby, British Columbia Canada

**Keywords:** Severe short stature, Floating-Harbor syndrome, Laron syndrome, *GHR*, *GHI*, *ACAN*, *SRCAP*

## Abstract

**Background:**

Linear bone growth is achieved by the division of chondrocytes at the growth plate and is regulated by endocrine and paracrine factors such as growth hormone. Mutations that negatively affect chondrogenesis can be a contributor to short stature. One such mutation can occur in the *ACAN* gene, causing short stature and advanced bone age. Similarly, mutations in growth hormone receptors (GHR) can lead to Laron syndrome (LS), one of the several disorders that are collectively called growth hormone insensitivity syndrome (GHI). Another example is Floating-Harbor syndrome (FHS), a rare autosomal dominant due to mutations in the *SRCAP* gene that can also result in short stature.

**Case presentation:**

We report the case of a 6-year-old female with concomitant mutations in the three genes mentioned above. The mutations reported here were found on genetic studies and are usually benign, causing a variant of undetermined significance. However, our patient’s phenotype could only be explained by the compounded effects of pathogenic mutations of these genes. Some of the same mutations were also found in the patient’s father and her paternal grandfather. Both also presented with short stature, though not to the same degree as our patient. While these mutations are often reported to be insignificant, they gave rise to severe short stature and a specific phenotype in the patient when presented together. We think that even though the GHI spectrum is inherited through an autosomal recessive pattern, the sum of these heterozygous mutations resulted in severe short stature despite the limited GHI seen in our patient, the father, and the grandfather, through a rare *ACAN* and *SRCAP* mutation that, to our knowledge, has not been previously reported as a pathogenic mutation in the literature.

**Conclusion:**

We investigated the possible synergistic effects of these variations on exacerbation or masking of the signs and symptoms of GHI with the hope of providing a better understanding of these genes and their function through our rare case.

## Background

Linear bone growth is achieved by chondrocyte division at the growth plate. Chondrocyte division is regulated by endocrine and paracrine factors such as growth hormone [1]. Mutations that negatively affect chondrogenesis can potentially be a contributor to short stature [1]. One such mutation occurs in the *ACAN* gene that encodes Aggrecan, which is a critical proteoglycan component of the extracellular matrix of the growth plate cartilage [2]. Most *ACAN* mutations are inherited through an autosomal dominant pattern and differ in phenotype, but all of them are characterized by short stature and advanced bone age [3–5].

Similarly, mutations in the growth hormone receptor (GHR) can lead to Laron syndrome (LS), one of the several disorders that are collectively called growth hormone insensitivity syndrome (GHI) [6–10]. Studies into heterozygous mutations have found that such mutations may indeed result in shorter-than-average stature, but not to what would be considered a pathological extent; as well, there have been limited studies to suggest that heterozygous mutations may result in GHI [11, 12]. While LS is an autosomal recessive condition, our investigation indicates that if there are simultaneous heterozygous mutations in different genes that normally contribute to linear bone growth, the occurrence of short stature with highly variable phenotypes is quite possible [10, 12–15].

Here, we report a case with three concomitant mutations in the aforementioned genes. Most of these mutations are of undetermined significance due to a lack of available research into these mutations. On the other hand, there is controversy as to the pathogenicity of the same variant of the *GHR* gene of our patient. However, our patient presented with a phenotype that could be explained by the compounded pathogenic effect of these mutations. Our aim in this report is to provide a better understanding of these mutations and to establish that they are likely pathogenic.

## Case presentation

### Clinical data

A 6-year-old girl presented at our clinic with short stature. She was born healthy at 38 weeks by vaginal delivery to non-consanguineous parents. At birth, she was 48 cm in height and weighed 2655 g with a head circumference of 34.5 cm. The pregnancy was uncomplicated. The subject had an Apgar score of 8 to 9. The patient’s father’s height was 155 cm, and her mother’s height was 153 cm. These values were respectively 2.1 and 0.6 standard deviations below average in their country of origin, Iran, when matched by age and gender.

On initial evaluation, the patient was of disproportionately short stature, 96 cm tall (− 3.5 SDS), and weighing 21.6 kg. Examination of the subject revealed facial features of long eyelashes, a wide columella, and a short philtrum, with no signs of early-onset puberty, and no evidence of congenital anomalies. Given the exam, an underlying genetic cause was suspected, but we aimed to rule out other possible causes through a complete medical evaluation that yielded the results presented below.

## Methods

Our investigations were performed in two phases: we firstly assessed the subject with a physical examination, blood tests, imaging, and genetic testing, and secondly, based on the results, genetic analyses of the subject’s parents were performed.

Blood tests were ordered to assess thyroid function, as well as insulin-like growth factor 1 (IGF1), growth hormone (GH), prolactin levels, and urinary AM cortisol; to rule out autoimmune-related malabsorptive diseases, anti-tissue transglutaminase antibody (anti-tTG) IgG, and IgA levels were evaluated. A GH stimulation test by clonidine was also performed. A urinalysis was conducted, as well as a renal ultrasound, and bone density was assessed. An X-ray was performed of her left hand/wrist to determine bone age. The national growth reference provided by Iran’s Ministry of Health and Medical Education was used in this study.

Subsequently, we ordered conventional G-banding karyotyping to screen for large duplications and deletions, balanced translocations, inversions, and ploidy changes. Then, for a more detailed analysis, we followed this with whole-exome enrichment, performed by the Agilent SureSelect V6 target enrichment kit on Illumina Hiseq 4000 platform with an average depth of 206X performed by Macrogen, South Korea. Nearly all exons and flanking 10 bp (base pairs) were detected and analyzed (detected variants included single-point mutations and small indels (within 20 bp)). The analytical sensitivity and specificity of the next-generation sequencing (NGS) method used in this assay for detection variants mentioned above was more than 95%, and nearly all exons were detected and analyzed.

Based on the results of our analysis of the subject’s genetic testing, we performed a polymerase chain reaction (PCR) followed by Sanger sequencing on the parents to further investigate the validity of the detected variants. This procedure was repeated on available relatives of the subject’s father (his father and his sister). All bioinformatics analysis of sequencing results was performed using international databases (American College of Medical Genetics, The Human Gene Mutation Database and ClinVar) and standard bioinformatics software.

After all evaluations had been completed, growth hormone therapy was initiated. The GH dose was increased to a maximum amount of 67 μg/kg per day for a course of 6 months of treatment.

### Clinical results

Analysis of the blood tests from the subject showed results within normal limits (WNL), although IGF1 levels were found to be at borderline low-normal limits (2.5th percentile) and GH levels were found to be mildly deficient (0.96 ng/ml), while GH stimulation was WNL. Urinalysis, prolactin, AM cortisol level, and bone density were all WNL, and the renal ultrasound was unremarkable. X-ray results were reviewed to determine bone age; her left hand/wrist X-ray showed results consistent with an age of 7 years when she was, in fact, 6 years old. Conventional G-banding karyotype showed no chromosomal abnormalities. Anti-tTG, IgG, and IgA were found to be WNL, suggesting no evidence of autoimmune-related malabsorptive disease.

Once all tests were concluded, GH therapy was initiated for the subject given her unsatisfactory growth velocity. With GH therapy, her growth velocity increased about 1.75 cm above the growth velocity prior to treatment. These results thus far point to treatment failure and partial insensitivity to GH.

### Molecular results

Results from whole exome enrichment showed that the subject was heterozygous for the *GHR* gene (NM_001242399.2 exon6: c.556C>T) that led to amino acid changes p.R186C, heterozygous for the *ACAN* gene (NM_013227.3 exon17: c.7418G>A) that led to amino acid changes p.R2473Q, heterozygous for the *SRCAP* gene (NM_006662.2 exon25: c.4259C>T) that led to amino acid changes p.S1420F, and heterozygous for the *AGBL1* gene (NM_152336.2 exon22: c.2969G>C) that led to amino acid changes p.C990S. These results are summarized in Table 1.

**Table 1:** The result of the patient’s genetic evaluation

Gene transcript (RefSeq)	Variant location	Variant	Chromosome position (GRCH37)	Type	Zygosity
*GHR* NM_001242399.2	Exon 6	c.556C>T p.R186C	Chr5:42.700.021	Missense	het
*ACAN* NM_013227.3	Exon 17	c.7418G>A p.R2473Q	Chr15:89.417.157	Missense	het
*SRCAP* NM_006662.2	Exon 25	c.4259C>T p.S1420F	Chr16:30.735.004	Missense	het
*AGBL1* NM_152336.2	Exon 22	c.2969G>C p.C990S	Chr15:87.217.553	Missense	het

To determine the origin of her mutations and to rule out any other variation in her family, WES was performed on the subject’s parents. Her father was heterozygous for the *GHR* gene (NM_001242399.2 exon6: c.556C>T) that led to amino acid changes p.R186C. Her mother was heterozygous for the *AGBL1* gene (NM_152336.2 exon22: c.2969G>C) that led to amino acid changes p.C990S. These results are summarized in Table 2.

**Table 2:** WES results of the subject’s parents

	Gene transcript (RefSeq)	Variant location	Variant	Zygosity	Type
Father	*GHR* NM_001242399.2	Exon 6	c.556C>T p.R186C	Het	Missense
Mother	*AGBL1* NM_152336.2	Exon 22	c.2969G>C p.C990S	Het	Missense

PCR amplification followed by Sanger sequencing for the variants c.556C>T p.R186C in *GHR* gene, c.2969G>C p.C990S in *AGBL1* gene, and c.7418G>A p.R2473Q in *ACAN* gene validated the variants detected via WES. The results are shown in Table 3.

**Table 3:** Results of PCR followed by Sanger sequencing

Sample	Gene/transcript	Chromosome position (GRCH37)	Variant	Zygosity
Patient	*GHR* NM_001242399.2	Chr5:42.700.021	c.556C>T p.R186C	het
Father	*GHR* NM_001242399.2	Chr5:42.700.021	c.556C>T p.R186C	het
Mother	*GHR* NM_001242399.2	Chr5:42.700.021	c.556C>T p.R186C	Normal homozygous
Patient	*AGBL1* NM_152336.2	Chr15:87.217.553	c.2969G>C p.C990S	het
Father	*AGBL1* NM_152336.2	Chr15:87.217.553	c.2969G>C p.C990S	Normal homozygous
Mother	*AGBL1* NM_152336.2	Chr15:87.217.553	c.2969G>C p.C990S	het
Patient	*ACAN* NM_013227.3	Chr15:89.417.157	c.7418G>A p.R2473Q	het
Father	*ACAN* NM_013227.3	Chr15:89.417.157	c.7418G>A p.R2473Q	Normal homozygous
Mother	*ACAN* NM_013227.3	Chr15:89.417.157	c.7418G>A p.R2473Q	Normal homozygous

To evaluate the effects for the variant c.556C>T p.R186C in the *GHR* gene, we ordered PCR followed by Sanger sequencing for the available family members of the patient’s father (his father and his sister). His father was heterozygous for the variant, and his height was 157 cm (− 1.8 SDS), nearly the same as his son; however, his sister, who was of average height at 165 cm (+ 1.1 SDS), did not inherit the variant.

Based on these results, we concluded that the patient’s father’s familial short stature could be due to his *GHR* mutation resulting in GHI spectrum manifestation. Although we posit that heterozygous mutations in the *GHR* gene are seen resulting in GHI despite having an autosomal recessive pattern of inheritance, we are not yet aware of how this is possible; so far, only the signs and symptoms concurrent with the mutations stand as evidence of their detrimental effects on height.

## Discussion

It is evident that our patient’s mutations in *ACAN* and *SRCAP* are sporadic and new, as they did not exist in her parents. Prior to GH therapy, the subject’s GH level was low while a GH stimulation test showed normal results; with GH therapy, the subject’s growth velocity did not increase to the degree expected, suggesting a partial insensitivity to GH.

As mentioned above, mutations in the *GHR* gene can result in GHI. The subject’s partial GH insensitivity is likely explained by her GHR mutation. Our patient had a heterozygous missense *GHR* mutation which previously has been reported as a pathogenic variant in The Human Gene Mutation Database (HGMD) and other studies [16], but the ClinVar Database does not support its pathogenicity [9]. Given that her father shares this mutation, it is highly likely that his short stature is also at least in part due to a similar GH insensitivity.

That said, the severity of our patient’s short stature is most likely the result of not only her GHR mutation, but the additive effects of her mutations in *ACAN* or *SRCAP* or indeed both these genes.

As explained earlier, pathogenic *ACAN* mutations can result in short stature and advanced bone age similar to those seen in our patient. However, to our knowledge, her specific mutation has not been reported as pathogenic in previous publications. Since her phenotype resembles those seen in pathogenic mutations of the *ACAN* gene, here we would like to suggest that her specific variation is likely pathogenic and a contributor to her advanced bone age and short stature.

Mutations in the *SRCAP* gene can result in Floating-Harbor syndrome (FHS), a rare autosomal dominant mutation in the *SRCAP* gene that can also result in short stature and other phenotypes like a triangular face, low hanging columella, short philtrum, wide mouth, and an elongated nose with a narrow bridge [17]. To our knowledge, the pathogenicity of our patient’s specific *SRCAP* mutation was not reported previously, and its significance is unknown; however, her long eyelashes, her seemingly wide columella, and her slightly short philtrum all resemble those futures seen in FHS, and could be the result of this mutation [18]. Since other work-up did not explain her mildly low levels of GH and IGF1, here we would like to report her *SRCAP* mutation as the cause; other studies have reported similar findings [19]. Mutations of the *AGBL1* gene are associated with late-onset of Fuchs’ corneal dystrophy, an autosomal dominant condition that results in corneal epithelial destruction [20]. In our patient, her mutation is likely pathogenic based on the American College of Medical Genetics and Genome (ACMG) and as reported in other studies [20]. Therefore, we referred our patient to an ophthalmologist to be further evaluated for this condition and to avoid corneal epithelial destruction and loss of eyesight.

The frequency in the general population, pathogenicity, signs, and symptoms of the aforementioned variants are summarized in Table 4.

**Table 4:** Variants along with frequency in the general population, based on genomAD; pathogenicity based on our study and ClinVar; and signs and symptoms attributed to the variant

Gene transcript (RefSeq)	Variant	Variant allele frequency (gnomAD)	Pathogenicity (ClinVar)	Zygosity	Attributed signs and symptoms	Pathogenicity based on our findings
***GHR*** **NM_001242399.2**	c.556C>T p.Arg186Cys	**0.003922**	Uncertain significance	**het**	short stature, partial insensitivity to growth hormone	**Likely pathogenic**
***ACAN*** **NM_013227.3**	c.7418G>A p.R2473Q	**0.0001071**	Undefined/unknown	**het**	advance bone age, short stature	**likely pathogenic**
***SRCAP*** **NM_006662.2**	c.4259C>T p.S1420F	**0.00001193**	Undefined/unknown	**het**	long eyelashes, seemingly wide columella, slightly short philtrum, mildly low levels of GH and IGF1, short stature	**likely pathogenic**
***AGBL1*** **NM_152336.2**	c.2969G>C p.C990S	**0.001130**	pathogenic	**het**	Undefined	**Undefined**

## Conclusions

To our knowledge, the concomitance of these mutations as they relate to the resultant phenotype in our patient is unique and lead to a new hypothesis: Based on our clinical investigations and our review of the latest relevant literature, the highly unusual phenotype seen in our patient can most likely be attributed to her particular variations.

One explanation for her phenotype is that these variants are pathogenic and of the autosomal dominant pattern of inheritance, despite being categorized in the VUS section. The evidence that we have to support our claim are the signs and symptoms of these variants that are observed only in pathogenic mutations of the genes, mentioned above. Regardless of the pattern of inheritance of these variations, the attributed signs and symptoms were mild, so we could assume that if they do cause short stature, it would be to a mild degree, in correlation with other signs and symptoms. That said, with short stature being one of the signs of all of these mutations, and given that its severity does not correlate directly with the other signs and symptoms, we can assume that ultimately the impact on stature is a result of the synergistic effects of all the abovementioned variations. One possible mechanism is that, along with her partial GHI, the mutation in her *SRCAP* gene caused reduced plasma levels of GH and IGF1, resulting in synergistic effects with the GHI. Likewise, the synergistic effects of *ACAN* and *SRCAP* mutations coinciding with GHI could explain our patient’s lack of response to treatment, in spite of her heterozygous *GHR* mutation. Thus, we can presume that while these particular variations may have an insignificant effect individually on a person’s height, their usual phenotypes could be altered due to the synergistic effects they impart on one another, influencing a patient’s height dramatically. Also, many patients are labeled as having idiopathic or familial short stature (the father of our patient is an example) because no genetic studies are conducted and they do not show signs or symptoms that could lead the physician to the correct diagnosis. Therefore, we think that many of these mutations are not as rare as previously believed, but frequently go undiagnosed due to their variable phenotypes. We suggest that a complete genetic study and WES may be beneficial in certain patients who are suspected to carry the aforementioned genes prior to assigning a diagnosis of idiopathic or familial short stature.

## Data Availability

The datasets used and analyzed during the current study are available from the corresponding author in response to reasonable requests and with the permission of the patient’s legal guardians.

## Abbreviations

Anti-tTG: Anti-tissue transglutaminase antibody
GHR: Growth hormone receptor
GHI: Growth hormone insensitivity syndrome
LS: Laron syndrome
FHS: Floating-Harbor syndrome
SDS: Standard deviations
IGF1: Insulin-like growth factor 1
HGMD: Human Gene Mutation Database
ACMG: American College of Medical Genetics and Genome
genomAD: Genome Aggregation Database
PCR: Polymerase chain reaction
NGS: Next-generation sequencing
het: Heterozygous
